# Vertical-cavity surface-emitting laser sources for gigahertz-bandwidth, multiwavelength frequency-domain photon migration

**DOI:** 10.1117/1.JBO.22.10.105001

**Published:** 2017-10-06

**Authors:** Thomas D. O’Sullivan, Keunsik No, Alex Matlock, Robert V. Warren, Brian Hill, Albert E. Cerussi, Bruce J. Tromberg

**Affiliations:** a University of California Irvine, Beckman Laser Institute and Medical Clinic, Laser Microbeam and Medical Program, Irvine, California, United States; b University of Notre Dame, Department of Electrical Engineering, Notre Dame, Indiana, United States; c Infit and Co. Inc., Seocho-gu, Seoul, Republic of Korea

**Keywords:** biomedical optics, lasers, medical imaging, sensors, spectroscopy, biophotonics

## Abstract

Frequency-domain photon migration (FDPM) uses modulated laser light to measure the bulk optical properties of turbid media and is increasingly applied for noninvasive functional medical imaging in the near-infrared. Although semiconductor edge-emitting laser diodes have been traditionally used as miniature light sources for this application, we show that vertical-cavity surface-emitting lasers (VCSELs) exhibit output power and modulation performance characteristics suitable for FDPM measurements of tissue optical properties at modulation frequencies exceeding 1 GHz. We also show that an array of multiple VCSEL devices can be coherently modulated at frequencies suitable for FDPM and can improve optical power. In addition, their small size and simple packaging make them an attractive choice as components in wearable sensors and clinical FDPM-based optical spectroscopy systems. We demonstrate the benefits of VCSEL technology by fabricating and testing a unique, compact VCSEL-based optical probe with an integrated avalanche photodiode. We demonstrate sensitivity of the VCSEL-based probe to subcutaneous tissue hemodynamics that was induced during an arterial cuff occlusion of the upper arm in a human subject.

## 1 Introduction

The frequency-domain photon migration (FDPM) technique, sometimes referred to as frequency-domain near-infrared spectroscopy, is used to measure the optical near-infrared (NIR) absorption and scattering properties of turbid media and is under investigation for noninvasive medical imaging.^1^^,^^2^ On living tissue, FDPM can provide quantitative functional biophysical information such as the deep tissue (1 to 2 cm) concentrations of chromophores (e.g., hemoglobin, water, and lipid) and the size and density of cellular and subcellular components. It has been applied to a wide-range of clinical applications including studies of the human brain,^3^^,^^4^ hemodynamic stress,^5^ muscle exercise,^6^ neonatal care,^7^ and breast tissue and tumors.^8^^–^^10^ FDPM separates light attenuation due to absorption and scattering by precisely measuring the amplitude and phase of an intensity-modulated light wave that has propagated through turbid media, also called a photon density wave. Light sources suitable for clinical FDPM systems typically require at least several milliwatts (mW) of optical power to be delivered to the tissue in the NIR (650 to 1000 nm) and modulated in the range of 50 to 1000 MHz. Both overall optical attenuation and source–detector separation affect the signal-to-noise ratio (SNR); thus, higher modulation depth (which is a function of both peak optical power and modulation bandwidth) is typically required for increased depth sensitivity and for penetrating higher attenuating tissues.

Vertical-cavity surface-emitting lasers (VCSELs)—semiconductor lasers that emit light normal to the substrate—have not been historically applied to FDPM techniques primarily because of their perceived low output power. However, modern, commercially available uncooled continuous-wave (CW) VCSELs output up to several hundred mW depending on wavelength (650 to 1100+  nm) and greater than 10 W in a two-dimensional array format. Originally intended for telecommunications applications, VCSELs are easily intensity-modulated at the frequencies relevant for optical property recovery. Their small size, simple packaging, and low power consumption make them particularly attractive for use in wearable devices and could enable new applications of quantitative diffuse optical spectroscopy. In this work, we show that single VCSELs and VCSEL arrays provide sufficient optical power, bandwidth, and modulation efficiency for broad bandwidth FDPM optical property measurements in solid tissue-simulating phantom experiments. We report a unique multiwavelength VCSEL-based FDPM source module with an integrated photoreceiver in a wearable format that is designed to be placed in direct contact with the tissue. We show, for the first time, to our knowledge, frequency-domain diffuse optical spectroscopy measurements of a human subject using a direct-contact VCSEL-based integrated optical probe.

## 2 Methodology

800-nm single-aperture and multiple aperture (2×2 array) VCSEL devices from Vixar Inc. (Plymouth, Minnesota) were evaluated in this study. A standard Fabry–Perot, single mode edge-emitting laser (EEL) diode was also tested for reference and comparison (LD808-SA60, Thorlabs Inc., Newton, New Jersey). All devices were mounted in 5.6-mm TO packages. Benchtop testing was performed by attaching the laser to a custom PCB with backside MMCX RF connectors to connect the high-frequency directly modulated laser drive current. The devices were current controlled without temperature stabilization and were tested at room temperature. For fiber-coupled measurements, the VCSEL output was collimated with an aspheric lens and coupled to a 400-μm core multimode silica fiber.

For modulation testing, the lasers were direct current modulated using a bias tee to combine DC current injection from a standard laser driver (LDC-3916370, ILX Lightwave, Bozeman, Montana) with an RF signal provided by a network analyzer (8753ES, HP/Agilent, Santa Clara, California). DC optical power was measured using an optical power meter (PM100USB, Thorlabs Inc., Newton, New Jersey), and AC modulation was measured using a high-speed (>2  GHz bandwidth) photodiode (UPD-200-SP, Alphalas, Göttingen, Germany). An RF switch (HMC253QS24, Hittite, Chelmsford, Massachusetts) enabled dynamic selection of the laser devices.

Tissue-simulating silicone-based phantoms with varying optical properties were measured using the VCSELs and EEL with the FDPM technique. Phantom construction has been described previously and was designed to simulate NIR optical absorption and scattering of human tissues.^11^ Optical properties were measured by placing a 1×1  mm circular active area avalanche photodiode (APD) module (S12060-10/C5658, Hamamatsu Corp., Shizuoka, Japan) on the surface of the phantom to collect multiply scattered photons 22 mm away from the source fiber. The network analyzer was used to measure the frequency-dependent amplitude attenuation and phase shift of the resulting PDW relative to the source. The instrument response was calibrated by first measuring another phantom with known optical properties. The optical absorption ($\mu_{a}$) and reduced scattering ($\mu_{s}^{\prime }$) properties were recovered by fitting a P1 approximation of the radiative transport model to the measured amplitude and phase data (50 to 500 MHz) with semi-infinite boundary conditions.^12^^,^^13^

**Fig. 1:**
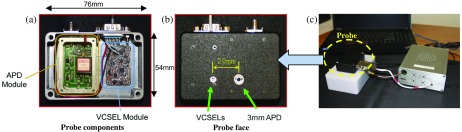
FDPM module and integrated probe containing a three-wavelength VCSEL package and 3-mm diameter circular APD. (a) The inside of the integrated probe consisting of a custom APD module with broadband transimpedance amplifier and high-voltage power supply. The VCSEL control module consists of digitally controllable switches and bias tees. (b) The probe face, which is placed in direct contact with tissue during operation. (c) The complete FDPM system, consisting the integrated probe, custom FDPM control module, and laptop computer for data collection and processing.

A custom broadband FDPM-module, the design and fabrication of which is described in Ref. 14, was modified to drive three VCSEL dies (680, 795, and 850 nm) in a single 5.6 mm package from Vixar, Inc. The VCSEL package was integrated into a handheld probe alongside a 3×3  mm circular active area APD (S6045-05, Hamamatsu Corp., Shizuoka, Japan), custom APD module, and laser (RF+DC) switching circuitry. Specifically, the handheld probe contains a custom APD module with broadband (50 to 500 MHz) transimpedance amplifier and high voltage power supply to bias APD. The VCSEL control module consists of digitally controllable switches and bias tees. All of the components required to operate the FDPM system include the integrated handheld probe, FDPM control module, and laptop computer for data collection and processing. The sources and detector were located 20 mm apart, and the overall probe was designed to be placed in direct contact with tissue. Amplitude and phase of the detected PDWs were measured by the custom FDPM-module and transferred to a laptop computer for analysis. The system components are displayed in Fig. 1.

## 3 Results

**Fig. 2:**
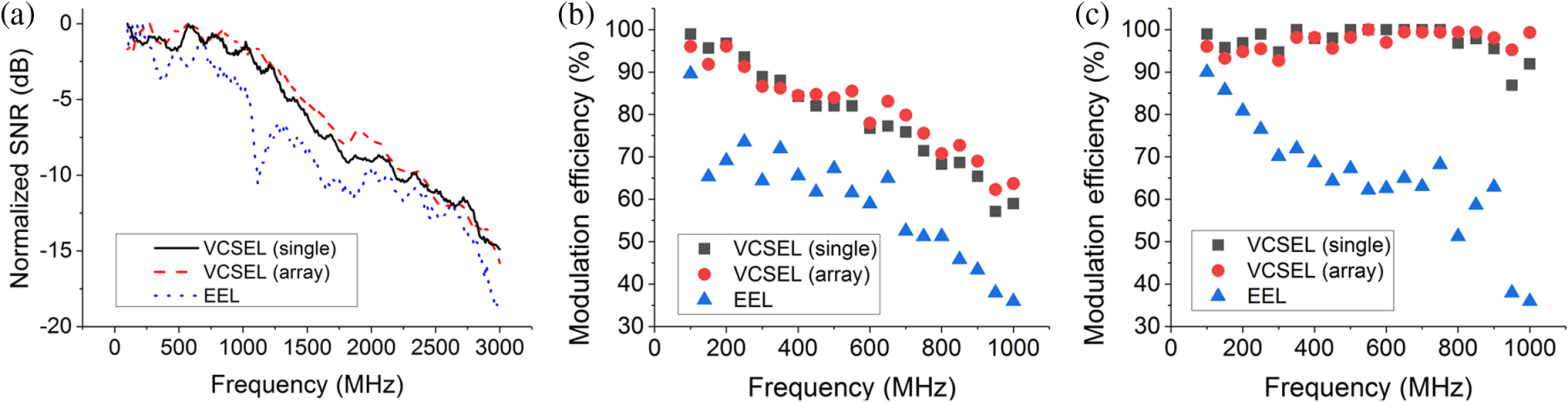
Modulation characteristics of the different laser types measured with a high speed photodiode. (a) Measured SNR at a fiber-coupled photodiode normalized to 0 dB at the frequency corresponding to the maximum SNR for each source. The SNR was calculated by dividing the detected RF power at each frequency by the detected power at a prior dark measurement. (b) Modulation efficiency for a fixed, frequency-independent RF injection power that was optimized at 100 MHz, and (c) maximum modulation efficiency before observable harmonic distortion.

Table 1 displays representative electrical and optical characteristics of the three evaluated lasers types: single aperture VCSEL, VCSEL array, and conventional EEL. For the VCSELs, performance was evaluated at the operating point corresponding to the maximum average optical power. Since the maximum optical power for the EEL (>100  mW) is beyond the Approved American National Standard (ANSI) recommended maximum permissible exposure for skin under CW operation,^15^ we chose to evaluate the EEL at an average optical power consistent with the ANSI limit for CW operation. While operating the EEL at lower optical powers similar to the VCSELs is possible, we chose not to because the nonlinear relationship between light output and driving current close to the threshold significantly distorts the laser modulation even at low RF injection powers. The peak wavelength of all of the lasers was measured to be 800±5  nm. Figure 2 shows the modulation characteristics of the three types of lasers. Figure 2(a) shows the detected SNR at a fiber-coupled photodiode when each laser was operated at a fixed DC bias and RF injection power over the modulation frequency range. The SNR was calculated by dividing the detected RF power at each frequency by the detected power at a prior dark measurement. To compare the frequency-dependent modulation characteristics and to remove the effects of different overall average optical power for each laser, the data were normalized such that the maximum SNR for each laser equals 0 dB. The differing frequency-dependent distortions among lasers in Fig. 2(a) are attributed to different impedance matching to the laser and its package. Figures 2(b) and 2(c) show the laser modulation efficiency as a function of modulation frequency, for fixed [Fig. 2(b)] and variable [Fig. 2(c)] RF injection power. Modulation efficiency was determined by examining the photodiode response on a 1-GHz bandwidth oscilloscope and calculating $(V_{\max}-V_{\min})/V_{\max}$. For Fig. 2(c), RF power was maximized to the point before harmonic distortion was qualitatively observed.

**Table 1:** Characteristics of the tested laser diodes and the optimal DC and RF bias selected to maximize intensity modulation depth at 50 MHz.

	Single aperture VCSEL	VCSEL array	EEL
Wavelength (nm)	801	795	798
Maximum DC optical power (mW)	6.3	11.8	>100
Optimal DC current at 50 MHz (mA)	22	45	70
Optimal RF power at 50 MHz (dBm)	13.0	17.7	10.7
Optical power at optimal bias (mW)	6.2	11.6	17.8
Total electrical power consumption (DC+RF) at optimal bias conditions (mW)	70	155	143
Ratio of power consumption to average optical power	11.1	13.4	8.0

**Fig. 3:**
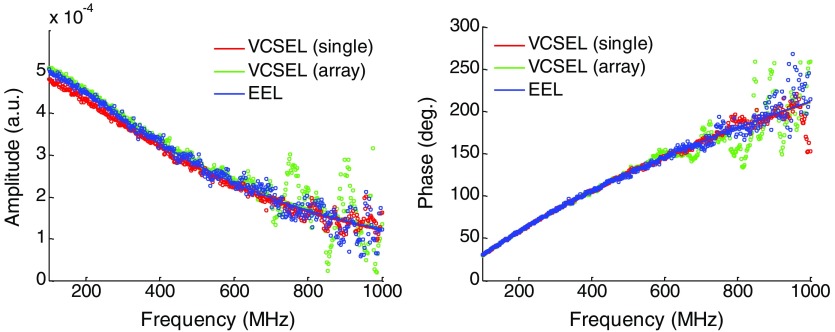
The calibrated amplitude and phase measured using FDPM through a tissue-simulating optical phantom ($\mu_{a}=0.007\;{\mathrm{mm}}^{-1}$, $\mu_{s}^{\prime }=1.01\;{\mathrm{mm}}^{-1}$) at a source–detector separation of 22 mm with a 1-mm-diameter circular APD.

**Fig. 4:**
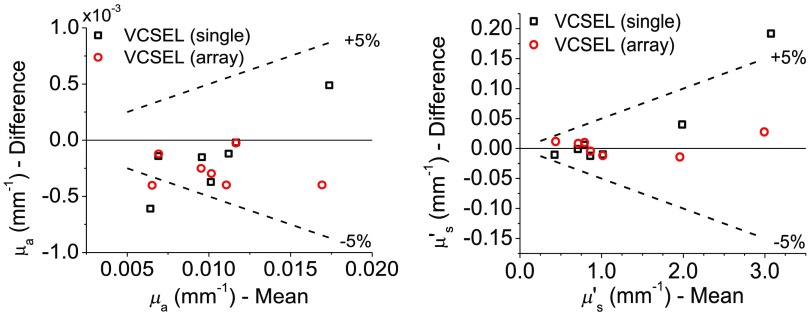
Bland–Altman plots that compare the optical properties recovered with the VCSEL devices (single and multiple aperture) with the standard EEL on seven different phantoms with varying optical properties. A 1-mm-diameter circular APD was used as the detector in all cases.

All three lasers were used to collect the frequency-dependent amplitude decay and phase shift of tissue-simulating phantoms at a source detector separation of 22 mm. The measured data and model fit on a single phantom are shown for all three devices in Fig. 3. The lasers were used to recover the optical properties of seven different tissue-simulating optical phantoms with a wide range optical properties that recapitulate different tissue types. Table 2 displays the optical properties of the tested phantoms as recovered by the standard EEL, and Fig. 4 compares the laser optical property recovery in Bland–Altman format. Between lasers, measured optical properties exhibited less than a 5% difference in most of the phantoms.

**Table 2:** Optical properties of the tested phantoms as measured using the standard EEL source.

Phantom #	$\mu_{a}$ (${\mathrm{mm}}^{-1}$)	$\mu_{s}^{\prime }$ (${\mathrm{mm}}^{-1}$)
1	0.0070	1.02
2	0.0096	0.71
3	0.0112	0.87
4	0.0117	1.97
5	0.0103	2.98
6	0.0067	0.79
7	0.0171	0.43

**Fig. 5:**
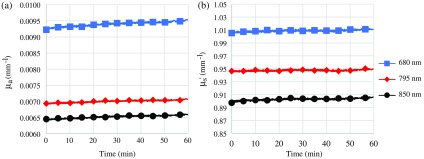
Data acquired with FDPM module and three-wavelength VCSEL/3 mm diameter circular APD integrated probe. (a) Measured optical absorption ($\mu_{a}$) and (b) reduced scattering ($\mu_{s}^{\prime }$) of a silicone-based tissue-simulation phantom measured continuously over 1 h (blue: 680 nm, red: 795 nm, gray: 850 nm).

Data collected with the VCSEL-based FDPM system are displayed in Fig. 5. Figure 5(a) shows the results of a 1-h continuous measurement in which the VCSEL probe was used to collect data every 3 s on a silicone tissue-simulating phantom. Optical properties were stable over this time period, as shown in Fig. 5. For 680 nm, $\mu_{a}$ (mean±SD) was $0.00094\pm 0.00007\;{\mathrm{mm}}^{-1}$ and $\mu_{s}^{\prime }$ was $1.009\pm 0.001\;{\mathrm{mm}}^{-1}$. This corresponded to a coefficient of variation (COV) of 0.69% and 0.13% for $\mu_{a}$ and $\mu_{s}^{\prime }$, respectively. The 795- and 850-nm VCSELs were stable as well, where $\mu_{a}$ was $0.00700\pm 0.00004\;{\mathrm{mm}}^{-1}$ (COV 0.49%) and $\mu_{s}^{\prime }$ was $0.947\pm 0.001\;{\mathrm{mm}}^{-1}$ (COV 0.10%) for the 795-nm VCSEL and $\mu_{a}$ was $0.00652\pm 0.00004\;{\mathrm{mm}}^{-1}$ (COV 0.65%) and $\mu_{s}^{\prime }$ was $0.903\pm 0.001\;{\mathrm{mm}}^{-1}$ (COV 0.15%) for the 850-nm VCSEL. There was a slight increase in $\mu_{a}$ over the 1 h measurement of 3.3%, 2.0%, and 2.2% for the 680, 795, and 850 nm VCSEL, respectively [Fig. 5(a)]. Less than 1.0% drift was observed for $\mu_{s}^{\prime }$ [Fig. 5(b)].

**Fig. 6:**
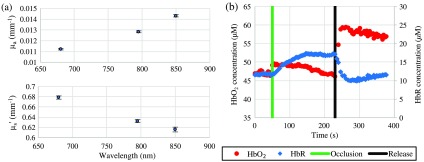
(a) Baseline tissue optical properties recovered at each VCSEL wavelength. Absorption coefficients are shown in the top plot and reduced scattering coefficients are shown in the bottom plot (mean ± standard deviation). (b) Recovered tissue concentrations of oxyhemoglobin (${\mathrm{HbO}}_{2}$, red) and deoxyhemoglobin (HbR, blue) from the arm during an arterial cuff occlusion. Green and black vertical lines represent the beginning and end of the arterial cuff occlusion, respectively.

To demonstrate the suitability for human measurements, we used the VCSEL module to monitor the subcutaneous hemoglobin concentrations in the underside of the forearm of a 32-year-old male subject during an upper arm arterial/venous cuff occlusion (cuff pressure=260  mmHg). The subject provided written informed consent, and data were collected under a human research protocol approved by the University of California Irvine Institutional Review Board (2004–3626). Baseline tissue optical properties at all three VCSEL wavelengths are shown in Fig. 6(a). Oxyhemoglobin and deoxyhemoglobin concentrations were calculated by fitting a linear combination of their known molar extinction coefficients^16^ to the measured absorption coefficients at the three VCSEL wavelengths. Tissue water and bulk lipid fractions were each assumed to be 35%, an estimate based on prior human forearm data collected with a broadband spectroscopy instrument. The water fraction is the concentration of measured tissue water divided by the concentration of pure water (55.6 M). The lipid percentage is the absorption of lipid measured relative to an assumed pure lipid density of $0.9\;g\;{\mathrm{mL}}^{-1}$. After baseline measurements were performed, the arterial cuff occlusion test was performed [Fig. 6(b)]. A manual inflation cuff that took several seconds to inflate was used for the occlusion. The inflation process occludes venous drainage prior to arterial delivery, which led to a small increase in ${\mathrm{HbO}}_{2}$ immediately during inflation of the cuff. During the 3 min of occluded blood flow, HbR increased ∼6  μM while ${\mathrm{HbO}}_{2}$ decreased ∼3  μM. Following release, trends reversed and a clear hyperemic response was observed.

## 4 Discussion and Conclusions

VCSELs are semiconductor laser light sources that are widely used for applications in telecommunications and sensing. They have extremely high bandwidths, having been used under direct current modulation to demonstrate optical links surpassing 40  Gb/s.^17^ Since laser light in VCSELs is emitted normal to the semiconductor wafer, they can be tested on the wafer without additional die cleaving or dicing. This significantly reduces the device cost.^17^ Mature GaAs-based VCSEL technology enables NIR laser sources that are ideal for tissue sensing in the biological window (650 to 1000 nm). Another benefit of vertical emission is that they can be easily and densely packaged into multicolor light source modules, which can benefit wearable devices. Both single aperture and array devices can be coupled to multimode optical fibers with high efficiency due to good mode matching.^18^^,^^19^ For these reasons, we hypothesized that VCSEL sources would be suitable optical sources, and in some cases better than EELs, for use in diffuse optical spectroscopy techniques such as FDPM. However, because of their small geometry, a single device’s maximum optical power output can be an order of magnitude or more less than the equivalent (single or multimode) EEL diode device. This low optical power combined with the extreme attenuation of light through tissue due to multiple scattering is the primary reason why VCSELs are not typically used in diffuse optical spectroscopy methods. Power can be increased using arrays of single VCSEL devices, but the suitability of combining multiple-aperture sources for FDPM has not been tested.

There are limited reports of using VCSELs in diffuse optical spectroscopy techniques, and a tunable-wavelength VCSEL-based device is now being commercialized for use in optical coherence tomography.^20^ Because of their small size and ease of integration, VCSELs have also been incorporated into miniature, rodent-worn CW near-infrared spectroscopy (NIRS) neural imaging systems^21^ and investigated for CW diffuse optical tomography.^22^ Sultan et al. first reported the use of multicolor VCSELs for FDPM optical property recovery through the simulation and fabrication of an optical source module designed for functional NIRS.^23^^,^^24^ Others have proposed and shown that pulsed VCSELs are suitable for time-domain optical spectroscopy.^25^^,^^26^ Due to self-heating effects that cause shifting of the VCSEL wavelength under high current injection, current modulation can be used to reduce speckle interference when VCSELs are used as a light source for diffuse optical imaging.^27^ In this paper, which is an extension of Ref. 28, we add to this body of work by including a detailed analysis of VCSEL performance that compares single-dies with higher power VCSEL arrays, compares VCSEL-sourced optical property recovery with standard EEL diodes, demonstrates a compact FDPM probe that integrates both VCSEL sources and a custom APD module, and reports a human measurement using a new, compact integrated VCSEL-based optical probe for use in direct contact with human tissue.

Our data demonstrate that single and multiple-aperture (array) VCSEL devices are capable light sources for FDPM recovery of optical properties in turbid media. We first examined the modulation characteristics to confirm that 800-nm VCSELs can modulate as well as EEL devices can. As expected, the VCSELs performed similarly to the EEL over the pertinent FDPM modulation range of 50 to 1000 MHz [Fig. 2(a)]. Modulation efficiency [Figs. 2(b) and 2(c)] was noticeably better at the selected biasing conditions for the VCSELs when compared with the EEL, possibly due to different impedances of the packaged devices. EEL modulation efficiency could be improved to nearly >90% similar to the VCSELs, but not without significant harmonic distortion. We expect that the VCSEL bandwidth would be equivalent or better than EELs in this range since frequency response will be limited by the parasitic limitations of the laser packaging and device contacts rather than the electro-optical dynamics of the laser cavity itself. At higher frequencies (10+GHz), due their higher intrinsic modulation bandwidth, properly designed and packaged VCSELs are expected to outperform EELs. Furthermore, we do not expect any differences in modulation performance at this frequency range between VCSELs of different wavelengths and confirmed this with the 680- and 850-nm VCSELs (data not shown).

We note that there was no difference in the modulation bandwidth of the single aperture VCSEL and the 2×2 multiple aperture VCSEL array, which suggests that VCSEL arrays are viable options for increasing optical power. We believe that, as long as it is possible to modulate the current required to drive higher power arrays, one can use VCSEL arrays to achieve even higher optical power levels as an FDPM source. While there may be a phase delay introduced in the RF modulation signal between apertures on one side of the array to the other (if the conductive lines were not designed with this in mind), the sum of these source signals will still result in single frequency overall modulation. This modulation may be delayed in phase slightly more than expected, and with a slightly lower average power due to interference, but this can be easily compensated for during standard instrument calibration procedures.

Table 1 shows that, of the devices tested, the EEL had the highest wallplug power efficiency under modulation. One may find this surprising as VCSELs are commonly touted as high power efficiency laser sources. This is true for some VCSEL devices, especially if the device structure is designed to optimize power efficiency. None of the devices that we evaluated for this work were designed for high efficiency, so we cannot make a broad claim as to which laser source is the most efficient for FDPM. However, it is important to note that overall power efficiency for FDPM will be a function not only of the DC wallplug power efficiency but also of the RF injection powered required for modulation and the modulation efficiency. These latter characteristics will be closely related to the RF impedance of the packaged device.

Next, we measured the optical properties of seven tissue-simulating phantoms with a range of attenuation using the single and multiple aperture VCSELs and the EEL. Using the EEL as a reference, VCSELs recovered the same absorption and reduced scattering coefficients within 5% in all but the most attenuating phantom (which is difficult to measure even for the standard EEL source).

Importantly, the small size and vertical-emission geometry of VCSELs enable simple integration of multiple dies in a single package. The miniature, dense integration of sources could be used to create FDPM imaging arrays as well as low-profile wearable FDPM sensors with more spectral density than using EELs alone. To demonstrate this benefit, we fabricated and tested a compact VCSEL-based FDPM probe. Similar to the size of a deck of cards, the probe contains a three-color VCSEL package, an APD, and the DC/RF electronics necessary to individually address the source channels—all controlled by a custom FDPM instrument module. The prototype system can collect FDPM measurements at a fixed 20 mm source–detector separation at frequencies from 50 to 500 MHz. The stability of the system, evaluated by measuring the optical properties of a fixed tissue simulation phantom over an hour, was equivalent to a large-format FDPM system ($<0.0010\;{\mathrm{mm}}^{-1}$ for $\mu_{a}$ and $0.01\;{\mathrm{mm}}^{-1}$ for $\mu_{s}^{\prime }$).^29^ This shows that uncooled VCSELs in this mode of operation may not need any additional thermal management. We hypothesize that the common increase in the absorption coefficient at all three wavelengths is largely due to self-heating of the APD module and the corresponding reduction in gain. However, we do note that there was slightly more drift in the absorption coefficient for the 680-nm VCSEL (3.3% versus 2.0% and 2.2% for the 795 and 850 nm VCSELs, respectively). This could be due to lower thermal conductivity in the 680-nm VCSEL structure.^30^ Because of the small active region, without appropriate heat sinking, the intensity of VCSELs can be noticeably more temperature dependent compared with EEL devices. Although our data suggest that thermal stability is not an issue for the tested VCSELs under these particular operating conditions, we would expect reduced thermal performance in devices that are not designed with good heat sinking or operated at high duty cycles. The lasing wavelength in VCSELs is known to vary less with temperature compared with EELs.^31^^,^^32^

To prove the suitability for biological tissue monitoring, the integrated probe was used to measure underarm subcutaneous hemodynamics during a cuff occlusion. We observed the expected hemoglobin deoxygenation during the occlusion, followed by a hyperemic recovery after the release. These two physiological phenomena are indicative of metabolism and vascular reactivity, respectively. Absolute values of hemoglobin concentrations before and during the occlusion agreed with expected values.^33^ Noninvasive, stable, cost-efficient, and portable methods to assess these phenomena, such as demonstrated in this work, could significantly impact health care once they are validated in clinical studies. To our knowledge, this is the first time that an integrated direct-contact optical probe containing VCSELs and an APD has been used to collect FDPM optical spectroscopy data from a human subject.

Recent demonstrations and ongoing development of electrically injected VCSELs that are dynamically tunable over wide wavelength ranges^34^^,^^35^ are particularly exciting for the future of tissue optical spectroscopy in a wearable format. Tuning is generally achieved by mechanically moving the top VCSEL mirror to shift the cavity resonance. The tuning range/spectral density performance of these devices cannot be achieved with an EEL-based device of comparable size. These tunable VCSELs have the potential to enable new sophisticated spectroscopy approaches in wearable devices without significantly increasing complexity, size, or cost, including broadband FDPM.

The optical source is only one of several components required in a wearable optical spectroscopy sensor. Additional major components include an optical detector and the electronics necessary to extract and transmit the time-resolved signals. Miniaturized integrated circuits to perform processing of FDPM data have already been demonstrated;^36^^,^^37^ however, detector miniaturization remains a challenge. Silicon-based APDs and photomultiplier tubes, common choices for FDPM measurements, both require a high voltage bias (>100 VDC) to achieve the required gain/sensitivity. However, recent advances in detector technology are promising in both reducing the voltage required and improving overall sensitivity.^38^

Overall, we conclude that VCSELs exhibit performance characteristics suitable for FDPM measurements of tissue optical properties. Their output power and modulation characteristics are more than sufficient for optical property recovery, and increased power can be achieved by employing an array of VCSEL devices. In addition, their small size, simple packaging, and modulation efficiency make them an attractive choice as components in clinical and next-generation wearable FDPM-based sensors.
